# A home-based, multidisciplinary liver optimisation programme for the first 28 days after an admission for acute-on-chronic liver failure (LivR well): a study protocol for a randomised controlled trial

**DOI:** 10.1186/s13063-022-06679-x

**Published:** 2022-09-05

**Authors:** Natalie LY Ngu, Edward Saxby, Thomas Worland, Patricia Anderson, Lisa Stothers, Anita Figredo, Jo Hunter, Alexander Elford, Phil Ha, Imogen Hartley, Andrew Roberts, Dean Seah, George Tambakis, Danny Liew, Benjamin Rogers, William Sievert, Sally Bell, Suong Le

**Affiliations:** 1 Department of Gastroenterology and Hepatology, Monash Health, Level 3, 246 Clayton Rd, Clayton, Victoria 3168 Australia; 2 Faculty of Medicine, Nursing and Health Sciences, Monash University, Wellington Rd, Clayton, Victoria 3800 Australia; 3 Hospital in the Home, Level 4, 246 Clayton Rd, Clayton, Victoria 3168 Australia; 4 Adelaide Medical School, The University of Adelaide, Corner of North Terrace & George St, Adelaide, South Australia 5000 Australia

**Keywords:** Cirrhosis, Interdisciplinary health team, Chronic disease

## Abstract

**Background:**

Acute-on-chronic liver failure (ACLF) represents a rising global healthcare burden, characterised by increasing prevalence among patients with decompensated cirrhosis who have a 28-day transplantation-free mortality of 33.9%. Due to disease complexity and a high prevalence of socio-economic disadvantage, there are deficits in quality of care and adherence to guideline-based treatment in this cohort. Compared to other chronic conditions such as heart failure, those with liver disease have reduced access to integrated ambulatory care services. The LivR Well programme is a multidisciplinary intervention aimed at improving 28-day mortality and reducing 30-day readmission through a home-based, liver optimisation programme implemented in the first 28 days after an admission with either ACLF or hepatic decompensation. Outcomes from our feasibility study suggest that the intervention is safe and acceptable to patients and carers.

**Methods:**

We will recruit adult patients with chronic liver disease from the emergency departments, in-patient admissions, and an ambulatory liver clinic of a multi-site quaternary health service in Melbourne, Australia. A total of 120 patients meeting EF-Clif criteria will be recruited to the ACLF arm, and 320 patients to the hepatic decompensation arm. Participants in each cohort will be randomised to the intervention arm, a 28-day multidisciplinary programme or to standard ambulatory care in a 1:1 ratio. The intervention arm includes access to nursing, pharmacy, physiotherapy, dietetics, social work, and neuropsychiatry clinicians. For the ACLF cohort, the primary outcome is 28-day mortality. For the hepatic decompensation cohort, the primary outcome is 30-day re-admission. Secondary outcomes assess changes in liver disease severity and quality of life. An interim analysis will be performed at 50% recruitment to consider early cessation of the trial if the intervention is superior to the control, as suggested in our feasibility study. A cost-effectiveness analysis will be performed. Patients will be followed up for 12 weeks from randomisation. Three exploratory subgroup analyses will be conducted by (a) source of referral, (b) unplanned hospitalisation, and (c) concurrent COVID-19. The trial has been registered with the Australian New Zealand Clinical Trials Registry.

**Discussion:**

This study implements a multidisciplinary intervention for ACLF patients with proven benefits in other chronic diseases with the addition of novel digital health tools to enable remote patient monitoring during the COVID-19 pandemic. Our feasibility study demonstrates safety and acceptability and suggests clinical improvement in a small sample size. An RCT is required to generate robust outcomes in this frail, high healthcare resource utilisation cohort with high readmission and mortality risk. Interventions such as LivR Well are urgently required but also need to be evaluated to ensure feasibility, replicability, and scalability across different healthcare systems. The implications of this trial include the generalisability of the programme for implementation across regional and urban centres.

**Trial registration:**

Australian New Zealand Clinical Trials Registry (ANZCTR) ACTRN12621001703897. Registered on 13 December 2021.

WHO Trial Registration Data Set. See Appendix 1

**Supplementary Information:**

The online version contains supplementary material available at 10.1186/s13063-022-06679-x.

## Main text

### Background

Acute-on-chronic liver failure (ACLF) is associated with a rising global healthcare burden, underpinned by increasing prevalence among patients with decompensated cirrhosis [1] and 28-day transplantation-free mortality of 33.9% [2]. A retrospective cost analysis of all ACLF unplanned admissions at our health service demonstrated a 30-day readmission of 34.4% and a 71.0% increase in total hospital costs between 2012 and 2018 [3]. There is evidence of increasing multimorbidity [4] in patients with cirrhosis, with an increasing prevalence of complications from comorbid non-alcoholic fatty liver disease and type 2 diabetes mellitus [5]. ACLF is associated with multi-organ failure, which requires a coordinated, multidisciplinary management approach [6]. Additionally, a high prevalence of socioeconomic disadvantage [7] impedes receipt of guideline-based treatments.

Compared to other conditions such as heart failure, those with ACLF have reduced access to integrated ambulatory care services [8] and care is fragmented through under-resourced outpatient clinics. Alcohol abuse is the most prevalent aetiology and independently contributes to the cirrhotic complications of malnutrition and sarcopenia due to inadequate oral intake of energy and protein, altered gastrointestinal absorption and protein/fat metabolism [9]. The combination of financial disadvantage, low health literacy, and active alcohol use creates a maelstrom of difficult-to-treat malnutrition and sarcopenia. The current model of ambulatory care for chronic liver disease is reactive, fragmented and lacks a robust framework for disease management. For new models of care to be adopted into complex healthcare systems, significant upfront costs need to be justified with high-quality evidence [10], which must address obstacles from patient, staff and healthcare system perspectives [11].

LivR Well is a multidisciplinary intervention aimed at reducing mortality and readmission through a home-based, liver optimisation programme. A prospective, single-arm feasibility study was conducted from March to October 2021 and enrolled 32 patients with ACLF (manuscript in progress). Participants received weekly medical review, nursing support, oral nutritional supplementation and consultation by dietetics, physiotherapy, pharmacy, social work and neuropsychiatry. The primary outcome was safety. Secondary outcomes were attrition, disease severity, healthcare utilisation, and patient-reported outcomes. Participants had a baseline median Charlson Co-Morbidity Index of 4 (IQR 3–5.5) and Model for End-Stage Liver Disease (MELD) score of16.5 (IQR 13–20.5). Handgrip strength in 11/23 (48%) met sex-specific sarcopenia criteria. Three patients died at 16, 148, and 197 days from enrolment. The 28-day mortality was 3% and the 30-day readmission rate was 18.75%. Median MELD score improved to 15 at 28 days (*p* = 0.16). The Chronic Liver Disease Questionnaire showed improvement in ‘fatigue’ (*p* = 0.02) and ‘worry’ (*p* = 0.03), at week 6. Patients, carers, and clinicians reported a positive experience in 100% and generated themes of acceptability, health literacy and insight, and autonomy. LivR Well cost $4947 AUD compared to $16,197 AUD for ACLF hospitalisation [3]. These results indicated that LivR Well was safe and acceptable, and likely to reduce both mortality and costs.

The feasibility study used the Asia-Pacific Association for the Study of Liver (APASL) definition of ACLF as an acute hepatic insult manifesting as jaundice (serum bilirubin ≥85mmol/L) and coagulopathy (International Normalized Ratio (INR) ≥1.5) complicated within 4 weeks by ascites and/or encephalopathy [12]. This study protocol for the RCT uses the European Foundation for the Study of Chronic Liver Failure criteria (page 5, Tables 1 and 2) [13], which grades the severity of ACLF using organ failure scores. Although the APASL definition was used due to our Asia-Pacific location, the decision was made to use EF-CLiF criteria to both identify and stratify the patients with the greatest risk of mortality, and to apply a definition most applicable to the Australian population with an alcohol misuse prevalence of 49.5% in cirrhosis [5] compared to greater chronic hepatitis B prevalence in the Asia-Pacific region at 2.8–7.6% [15] vs. 0.5–0.7% in Europe [16]. Consequentially, the patient population identified using EF-CLiF criteria are anticipated to have greater healthcare requirements and mortality but represent only a proportion of those admitted to hospital with decompensated liver disease. This protocol for a randomised controlled trial (RCT) therefore includes two arms to the trial: (a) those with ACLF and (b) those with hepatic decompensation not meeting EF-CLiF criteria. The RCT aims to provide a robust evaluation of the cost-effectiveness and impact on mortality and re-admission rates in each of these two populations compared to standard care.

**Table 1:** ACLF Grading using CLIF-C ACLF criteria [13, 14]

ACLF grade	Criteria
**No ACLF**	No organ failure or One organ failure (liver, coagulation, circulatory, respiratory) with serum creatinine <1.5mg/dL and no HE or Single cerebral failure and serum creatinine <1.5mg/dL
**Grade 1**	Single kidney failure or Single liver, coagulation, circulatory, or respiratory failure + serum creatinine 1.5–1.9mg/dL and/or HE I–II or Single cerebral failure (HE III–IV) + serum creatinine 1.5–1.9mg/dL
**Grade 2**	2 organ failures
**Grade 3**	3 or more organ failures

*mg/dL* milligrammes per deciliter, *HE* hepatic encephalopathy

**Table 2:** Defining organ/system failure using the CLIF Consortium Organ Failure Score (CLIF-OF) [14]

Organ system	Parameter	Score=1	Score=2	Score=3
**Liver**	Serum bilirubin (mg/dL)	<6	6≤12	>12
**Kidney**	Serum creatinine (mg/dL)	<2	2<3.5	≥3.5 or renal replacement therapy
**Brain**	West-Haven Grade	0	I-II	III-IV
**Coagulation**	INR	<2.0	2.0<2.5	≥2.5
**Circulation**	MAP (mmHg)	≥70	<70	Vasopressors
**Respiratory**	PaO2/FiO2	>300	≤300 and >200	≤200
	OR SpO2/FiO2	>357	>214 and ≤357	≤214

*mg/dL* milligrammes per deciliter, *INR* International Normalized Ratio, *MAP* Mean arterial pressure, mmHg millimetres of mercury, *PaO2* Partial pressure of arterial oxygen, *FiO2* Fraction of inspired oxygen, *SpO2* pulse oximetric saturation

### Methods/design

#### Aims

1. To evaluate the impact of the programme on 28-day mortality and 30-day re-admission
2. To model the cost-effectiveness of LivR Well compared to usual ambulatory care
3. To examine the differences in outcomes between those with ACLF and those with hepatic decompensation alone

#### Outcomes

##### Primary outcomes

- ACLF cohort: 28-day mortality
- Hepatic decompensation cohort: 30-day re-admission

Secondary outcomes

- Changes in liver disease severity using Model for End-stage Liver Disease Score and Child-Pugh class assessed at day 28 and week 12 compared to baseline
- Quality of life using Chronic Liver Disease Questionnaire and EQ-5D at week 6 compared to baseline
- Cost-effectiveness compared to standard care.

#### Design

The RCT will be a multi-site, non-blinded, parallel-group study with two independent population arms to determine the efficacy and safety of the new model of care (LivR Well) compared to standard care and to inform a cost-effectiveness analysis.

This study protocol was developed in line with Recommendations for Interventional Trials (SPIRIT) guidelines including the SPIRIT checklist [17], which has been adapted in accordance with World Health Organisation registration (Appendix 1) and the CONSORT extension to pilot and feasibility trials [18] (Table 3, Appendix 2).

**Table 3:**
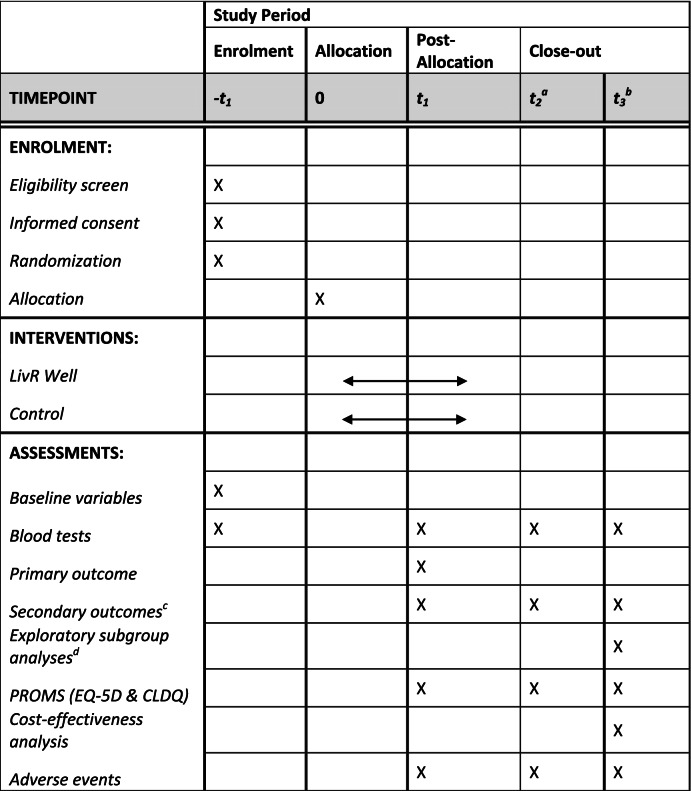
Standard Protocol Items: Recommendations for Interventional Trials (SPIRIT) figure

t_1_ 28 days after allocation, t_2_^a^ week 6 follow-up, t_3_^b^ week 12 follow-up

^c^Changes in liver disease severity, quality of life, and cost-effectiveness compared to standard care

^d^(a) Source of referral, (b) re-enrolment after unplanned hospitalisation, (c) COVID-19, and (d) hepatic decompensation without ACLF

#### Participants and setting

The study context is a multicentre tertiary health service in Melbourne, Australia with recruitment from three tertiary hospital campuses. Monash Health is the second largest healthcare service in Australia managing a catchment population greater than 1.2 million people [19], which includes the largest Culturally and Linguistically Diverse (CALD) community, the significant burden of socioeconomic disadvantage and the highest unemployment rates in the state of Victoria [20]. The 28-day intervention will be delivered through ‘Hospital in the Home’ (HITH), a medically-led ambulatory care programme supported by physical clinics and home visits by the nursing team. HITH is able to deliver many aspects of acute hospital care in the community to otherwise stable patients including diagnosis and management of acute conditions that would typically require bed-based care

Patients will have access to up to three home visits per week by nursing staff and physiotherapists. The patients will attend a hospital-based ambulatory care liver clinic weekly for a medical and nursing review with a dietitian, social work, addiction medicine, and pharmacist consultations as required. Patients will be referred to LivR Well from an outpatient context, the emergency department or the inpatient ward. Patients with any severity of liver disease are eligible unless receiving end-of-life care. There are no cut-offs for the MELD score or Child Turcotte Pugh score.

#### Inclusion criteria

##### Adult patients

- with previous or current hepatic decompensation defined as presence of ascites, hepatic encephalopathy, and/or variceal haemorrhage [21].
- with or without a diagnosis of acute-on-chronic liver failure (ACLF) using the European Foundation for the Study of Chronic Liver Failure criteria (EF-CLIF) including age and white cell count. Severity is graded according to the number of organ failures (Tables 1 and 2) [14].
- Requiring consultation from ≥3 allied health clinicians (Table 4)

**Table 4:** Allied health referrals

Clinician	Indication for referral	Tasks
Physiotherapist	Sarcopenia^a^, fall within last 6 months, FRAT^b^ score >11	6-min walk test (baseline and at day 28) Weekly, supervised low-intensity weight and resistance exercises
Dietitian	Sarcopenia, diabetes, alcohol dependence, MUST^c^ score ≥2	High protein, high energy, low salt diet plan incorporating compact nutritional supplements including a late-night snack
Social Work	Requiring long-term home support services, established disability, age >65 years	Referral for council services, aged care assessment, disability
Neuropsychiatry	Concern from the medical team regarding cognition	Neuropsychiatric assessment
Pharmacist	Polypharmacy (≥5 medications daily) and/or requiring titration of diuretic or lactulose doses	Patient and carer education, liaising with community pharmacy, organise blister pack
Addiction Medicine	Substance use disorder and alcohol pharmacotherapy	Weekly consultations, pharmacotherapy to manage substance use disorders

^a^Sarcopenia is defined using the European Working Group on Sarcopenia in Older People (EWGSOP) hand grip strength cut off (<27kg for males, <16kg for females) [22]

^b^ *FRAT* Falls Risk Assessment Tool [23]

^c^ *MUST* Malnutrition Universal Screening Tool [24]

##### Defining ACLF

- The diagnosis and grading of ACLF will be performed using the publically-available online calculator incorporating this formula: 10×[0.33×CLIF-OFs + 0.04×Age + 0.63×Ln (White cell count) − 2] [13]. (Tables 1 and 2)
- SI units of measurement (μmol/L) will be converted to mg/dL for serum bilirubin and serum creatinine for use in the calculator.

#### Exclusion criteria

- Greater than grade 2 hepatic encephalopathy
- Severe chronic extrahepatic disease
- Active malignancy including hepatocellular carcinoma
- Inability to provide informed consent
- Residing outside the local hospital service catchment or deemed ineligible for home visits due to staff safety or occupational hazard concerns
- Residing in a residential aged care facility

N.B. Concurrent COVID-19 infection is not an exclusion criterion.

##### Prohibited concomitant care

- Admission for scheduled procedure or treatment
- Moribund or receiving end-of-life care
- Receiving regular albumin infusions for the treatment of diuretic refractory ascites or chronic hepatorenal syndrome (excluding those for periprocedural circulatory support following large volume paracentesis)
- Refractory ascites managed with an intra-peritoneal catheter in-situ

#### Processes, interventions and comparisons

LivR Well is a 28-day intensive liver optimisation programme delivered at home and through the outpatient clinic. It is an innovative multidisciplinary model of care co-developed with patients and carers including delivery system re-design (home-based nursing, weekly medical reviews, regular dietitian and pharmacy consults) and adjunct interventions including enteral nutrition for sarcopenia, physiotherapy, large volume paracentesis, addiction medicine consultation and neuropsychiatric assessment for cognitive impairment/hepatic encephalopathy. Referrals to allied health clinicians will be made based on indications outlined in Table 4.

**Fig. 1:**
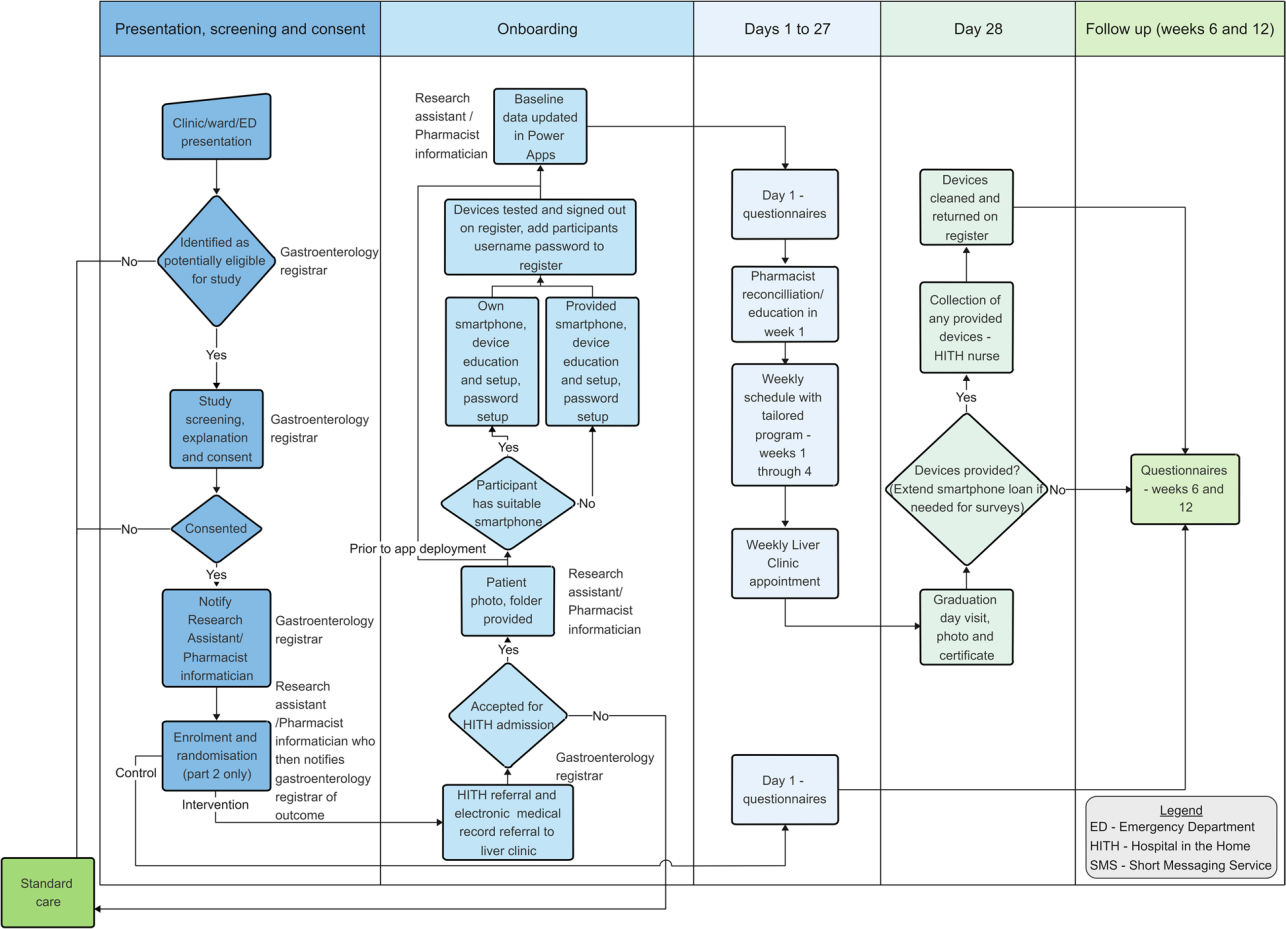
Patient journey map

Patients will be enrolled on discharge from the acute admission, directly from the emergency department or the outpatient clinic as demonstrated in the Patient Journey Map (Fig. 1). A standardised checklist template (Appendix 3) will be completed on admission. Patients will have an in-home clinical review by a nurse up to three times per week with observations and blood tests taken weekly, or as requested by the clinic gastroenterologist. A face-to-face appointment at the ambulatory complex care liver clinic will occur weekly and will provide medical and nursing reviews with the opportunity for a dietitian, pharmacist and social worker consultations as required. Key time points and tasks for completion are outlined in Table 5.

**Table 5:** Key time points and tasks for completion

	Day 1	28-day programme	Week 6	Week 12
Timeframe for completion	Within 3 days	Weekly	Within 7 days	Within 2 weeks
Tasks	Blood tests EQ-5D CLDQ	Blood tests Complex Care Liver Clinic	Blood tests DQ-5D CLDQ	Blood tests Complex Care Liver Clinic

Patients on the LivR Well programme will be remotely monitored in between clinic appointments through a custom-built app paired to a Bluetooth scale. Patient-reported outcome measures (PROMS) including quality of life, will be collected through a mobile phone text message link to the online surveys. Patients will also be able to access Lucy LiverBot, a custom-built artificial intelligence ChatBot for all disease-related questions. Quality of life assessments will be performed at day 1 and longitudinally at 6 weeks and 12 weeks from admission in both arms of the RCT. Two validated instruments will be used [EuroQol 5 Dimensions (EQ-5D) [25] and Chronic Liver Disease Questionnaire (CLDQ)] [26]. Outcomes from these assessments will guide the economic evaluation for a cost-effectiveness analysis. Adherence will be monitored through recording attendance at appointments, home visits and regular urinary ethyl glucuronide tests, a biomarker of alcohol consumption [27].

The comparator will be outpatient management in a ‘complex care liver clinic’, an ambulatory care clinic for patients with decompensated cirrhosis, promoting continuity of care by a gastroenterologist, hepatology nurse and pharmacist through regular face-to-face appointments. The control cohort will have no access to the additional allied health clinicians, technology aids or home visits through the LivR Well programme.

#### Recruitment

Patients will be recruited from the following settings: (a) following an acute hospital admission, (b) directly from the emergency department if able to be discharged home or (c) from the complex liver care clinic if ACLF or decompensation develops. All patients will be assessed by a gastroenterologist and allied health clinicians prior to recruitment. A research assistant or Pharmacist Informatician will provide eligible patients with a Participant Information and Consent Form (PICF) (Appendix 4) and consent will be obtained by a sub-investigator. Patients will be reviewed and admitted to the programme on the day of enrolment. All patients receiving LivR Well will also be onboarded to a mobile application and provided with the requisite hardware and software. Participants will be discontinued from the trial if consent is withdrawn, or if a serious adverse event (as defined on page 9) or death occurs. All participants will be included in the intention-to-treat analysis. If patients are admitted to the hospital after enrolment but before completion, the 28-day programme can be re-started on discharge if the patient still meets eligibility criteria and at the discretion of the treating team.

#### Screening and randomisation

Patients will be screened against inclusion and exclusion criteria at the time of diagnosis of ACLF or hepatic decompensation. Those who meet the criteria will be enrolled into the study and provided informed consent. These recruited patients will be randomised in a 1:1 ratio to either the LivR Well programme or to complex care liver clinic in the control arm. Participants will be computer randomised and informed of their allocation by the CLD nurse specialist prior to discharge or within 24 hours of obtaining informed consent. Patients from both study arms will be registered on the Virtual Hospital masterlog through the Microsoft Power Apps program. Due to the nature of the intervention, blinding of study participants or clinical staff administering the LivR Well programme will not be possible. However, outcome ascertainment for the primary outcome, as well as the health services utilisation secondary outcomes will be blinded. Furthermore, all data analyses will be undertaken in a blinded manner. The statistician will be blinded to the feasibility study data results and the treatment allocation of the RCT.

#### Trial status

We anticipate recruitment at a rate of 1-2 participants per week based on the progress of the feasibility study conducted at the same health service. A participant flow chart describing patients from screening, to enrolment, to randomisation, and allocation will be provided and will demonstrate how many patients are eligible for the intervention out of the entire study population.

#### Data collection

All data will be de-identified at the collection and stored securely on a Power BI database. Recruitment, retention and questionnaire completion numbers will be recorded throughout the trial. Biological specimens will be collected for the study and for patient care and analysed and stored through standard hospital processes. Consent will not be obtained for specimens to be used for genetic, molecular analysis, or in future/ancillary studies.

Data collected will include

- Demographic information including age, sex, primary language
- Clinical data at baseline, 28 days and 6-week follow-up
- Patient-reported outcome measures using EQ-5D and CLDQ quality of life instruments
- Hospital admissions, referral for liver transplantation and death

#### Adverse events

Adverse events are defined as “any untoward medical occurrence in a patient or clinical trial participant”. Serious adverse events (SAEs) will be defined as …” an untoward occurrence that results in death; is life-threatening; requires hospitalisation or prolongation of existing hospitalisation; results in persistent or significant disability or incapacity; consists of a congenital anomaly or birth defect” [28]; and will be reported to the PI and local ethics committee immediately. Any patients suffering harm from the trial will receive post-trial care in the liver clinic. Adverse event and SAS data will be collected in the same Power BI database at the time of occurrence by the hepatology nurse consultant.

#### Interim analysis

A data monitoring committee will consist of 3 senior independent clinician researchers without competing interests. An interim analysis will be conducted and we will adopt a Bayesian approach for an adaptive trial design where efficacy and futility criteria will be based on the probability of treatment effects, given the observed data in our feasibility study. One interim analysis will be performed in the ACLF and decompensated cohort when 50% of patients have been randomised and have completed 12 weeks of follow-up. We will stop the RCT for efficacy if the posterior probability (Pr) that the hazard ratio (HR) is < 1 is > 90% (Pr (HR <1|data) > 0.9. Similarly, we would stop for futility if Pr (HR <1|data) < 0.1. The results are reviewed by the independent data monitoring committee who will ultimately guide the decision to continue or cease the study.

#### Sample size

Sample size calculations were performed for each of the ACLF and hepatic decompensation arms using a 2-sided alpha of 5%, a power of 80%, and an anticipated 20% dropout.

For the ACLF cohort, we used the primary endpoint 28-day mortality, based on published rates of 22–74%, with a rate of 33.9% demonstrated in a study of 1343 hospitalised patients at 29 liver units in 8 European countries [2]. A 75% reduction in mortality in the intervention group generates a mortality rate of 8.5%, a conservative estimate considering our feasibility study of 59 participants resulted in a 3% 28-day mortality rate. We calculate a total sample size of 120 participants or 60 in each arm for the ACLF cohort. For the hepatic decompensation cohort, the sample size calculation is based on the primary endpoint of 30-day re-admission, which is estimated 17–38% [3, 29, 30]. Re-admission rates in our feasibility were 50% lower than anticipated at 18% by 30-days therefore we used an anticipated 17% 30-day re-admission rate in the intervention arm. We calculate a total sample size of 320 or 160 in each arm for the hepatic decompensation cohort. . Sample size calculation was performed using Stata statistical software, version 16.1 for Mac (StataCorp, College Station, TX, USA).

#### Statistical analyses

The data analysis for the primary composite endpoint of death, referral for liver transplantation or 30-day readmission from the date of hospital discharge will be undertaken on an intention-to-treat basis. A 28-day mortality rate will be calculated from the date of randomisation. For the primary and other binary outcomes, chi-squared tests will be used to compare the intervention and control groups. Survival analyses will also be undertaken, with the generation of Kaplan-Meier curves and Cox regression analyses to derive (adjusted) hazard ratios. For continuous outcomes, t-tests or non-parametric equivalents (e.g., Mann-Whitney tests) will be applied to compare the intervention and comparator groups. A *p* value of 0.05 is considered significant and 95% confidence intervals will be reported for all effect estimates Three exploratory subgroup analyses are planned: (a) comparing groups by the source of referral—conducted to address bias created following randomisation whereby in-patients in the intervention arm may be discharged home earlier due to perceived safety with regular home visits compared to those in the control arm. This subgroup analysis will account for differences between those admitted from in-patient admission compared to the emergency department or the ambulatory liver clinic; (b) comparing groups by re-enrolment to LivR Well after drop out due to unplanned re-admission to hospital. Data from the first enrolment to LivR Well will be included in the primary outcomes on an intention-to-treat basis and (c) patients with COVID-19 at the time of referral. Analyses will be performed using Stata statistical software, version 16.1 for Mac (StataCorp, College Station, TX, USA).

#### Economic analysis

The economic analysis will examine the cost-effectiveness of the LivR Well programme compared to the less resource-intensive control arm and will include both health system and societal cost perspectives. The main output of interest is the incremental cost-effectiveness ratio (ICER) in terms of net costs per unit of health gain. Health gains will be measured in clinical outcomes, estimation of years of life gained, quality-adjusted life years (QALYs) gained and by collection of quality-of-life data. Net costs will comprise the differential costs of the LivR Well programme and usual care minus costs saved from reduction in downstream health service utilisation. All health economic analyses will be undertaken in accordance with recommended approaches such as 5% discounting of estimated future costs and health gains. To account for any uncertainty in the data inputs for health economic modelling, sensitivity and uncertainty analyses will be undertaken via Monte Carlo simulation [31].

#### Dissemination of trial results

Trial results will be disseminated to healthcare professionals and the public through publication in a peer-reviewed journal and presentation at national and/or international conferences. Results will be distributed to participants during post-trial follow-up in the liver clinic. There are no specific publication restrictions; however, a high-impact hepatology journal will be approached.

## Discussion

We will evaluate the feasibility, acceptability, safety, efficacy and cost-effectiveness of LivR Well, a 28-day multidisciplinary liver optimisation programme following hospital admission with ACLF or hepatic decompensation. The programme consists of regular home visits, weekly medical reviews and access to multi-disciplinary team (MDT) care including medical, nursing, pharmacy, dietetics, social work and physiotherapy. Our feasibility study of 32 patients found that the programme was safe, feasible and acceptable and demonstrated a mortality rate of 3% and a re-admission rate of 18.5%.

The single-centre RCT aims to evaluate the impact on mortality and re-admission rate as well as disease severity, quality of life and cost-effectiveness. We anticipate that these results will guide the application of the programme in our quaternary health service, in other similar services and potential adaptation for use in regional centres.

One of the key strengths of this intervention is the use of a MDT approach enabled by technology. The MDT approach has previously been demonstrated to reduce mortality and outcomes in other chronic diseases including heart failure and chronic obstructive pulmonary disease [32]. The additional contributing factors including psychosocial issues associated with alcohol and other substance use disorders that contribute to poor health outcomes in this population must be acknowledged and addressed. The holistic approach of existing chronic disease management strategies can be applied to this similarly complex cohort. A nurse-led chronic disease management programme was assessed by Wigg et al. [8] in 60 patients randomised to the intervention (n=40) or usual care (*n*=20), which did not demonstrate an improvement in hospital admission rates, disease severity or patient quality of life. However, that study was limited by the sample size and did not incorporate a MDT. Our study aims to further explore these outcomes using a MDT approach, the inclusion of patients during an acute deterioration and with a larger population.

We acknowledge limitations in this study design and the ways we have attempted to address these. The trial is unblinded by nature, which may influence decisions such as referral for liver transplantation, re-admission to and discharge from hospital, clinical decisions especially for the control group, and referral for other procedures eg elective surgery. The statewide liver transplantation unit is external to Monash Health therefore referrals for consideration of transplantation are made using the centre’s own criteria. The other decisions will be challenging to address the influence of knowledge of the allocation group, and as such we will include this in the discussion. Patients in the intervention group may be re-admitted to the hospital faster due to close follow up or conversely, patients in the control group may be re-admitted at a lower threshold due to the absence of home-based support.

The economic evaluation will be a cost-effectiveness analysis and is essential to guide health services to strategically invest in sustainable ambulatory care to reduce emergency presentations and improve healthcare-related quality of life. This is of particular importance in this population with a high healthcare system burden [33], re-admission [34], and mortality rates.

The subgroup analyses are considered ‘exploratory’ as we acknowledge the potential for bias and challenging interpretation of results. It is unlikely with our total sample sizes that the subgroups will be adequately powered to draw conclusions and therefore another consideration could be using these data in a sensitivity analysis.

### Trial status

Study protocol V15

The trial is open for recruitment from January 10, 2022. The approximate date for completion of recruitment is March 2023

## Supplementary Information

**Additional file 1.** WHO Trial Registration.

**Additional file 2.** SPIRIT Checklist.

**Additional file 3.** LivR Well standardised admission checklist.

**Additional file 4.** PICF.

## Data Availability

On completion of the trial, the final datasets generated and/or analysed will be available from the corresponding author on request. Access to the final datasets will be in accordance with governance policies, Good Clinical Practice guidelines and ethics committee policies. The research team will be accountable to an independent data and safety monitoring board throughout the study. Any protocol amendments will be communicated to all relevant parties.

## Abbreviations

ACLF: Acute-on-chronic liver failure
CLD: Chronic liver disease
CLDQ: Chronic Liver Disease Questionnaire
EQ-5D: Euro Quality of Life 5 Dimension
FiO_2_: Fraction of inspired oxygen
FRAT: Falls Risk Assessment Tool
HGS: Handgrip strength
HITH: Hospital in the Home
INR: International Normalized Ratio
MAP: Mean arterial pressure
MUST: Malnutrition Universal Screening Tool
MDT: Multidisciplinary team
MELD: Model for End-Stage Liver Disease
PaO_2_: Partial pressure of arterial oxygen
PI: Primary investigator
PICF: Patient informed consent form
PROM: Patient-reported outcome measures
RCT: Randomised controlled trial
SAE: Serious adverse event
SPIRIT: Standard Protocol Items: Recommendations for Interventional Trials
SpO_2_: Pulse oximetric saturation
WHO: World Health Organization
