# Supplementary material for: A home-based, multidisciplinary liver optimisation programme for the first 28 days after an admission for acute-on-chronic liver failure (LivR well): a study protocol for a randomised controlled trial
Source: Trials. 2022 Sep 5;23:744. doi: 10.1186/s13063-022-06679-x (PMC9444080; doi:10.1186/s13063-022-06679-x)
Supplement: Supplementary file 1 — Additional file 1. WHO Trial Registration. [file 13063_2022_6679_MOESM1_ESM.docx]

# WHO Trial Registration Data Set (TRDS)

A home-based, multidisciplinary liver optimisation program for the first 28 days after an admission for acute-on-chronic liver failure (LivR Well): a protocol for a randomised controlled trial

1. Primary registry and Trial Identifying Number
   1. Australian New Zealand Clinical Trials Registry (ANZCTR)
      Trial ID: ACTRN12621001703897
2. Date of registration
   1. 13/12/2021
3. Secondary identifying numbers
   1. N/A
4. Sources of monetary or material support
   1. N/A
5. Primary sponsor
   1. N/A
6. Secondary sponsors
   1. N/A
7. Contact for public queries.
   1. A/Prof Suong Le
8. Contact for scientific queries
   1. A/Prof Suong Le (Principal Investigator)
9. Public title
   1. LivR Well
10. Scientific title
    1. A home-based, multidisciplinary liver optimisation program for the first 28 days after an admission for acute-on-chronic liver failure (LivR Well): a protocol for a randomised controlled trial
11. Countries of recruitment
    1. Australia only (single-centre)
12. Health condition studied
    1. Decompensated chronic liver disease
13. Intervention
    1. LivR Well – a coordinated, home-based multidisciplinary 28 day program for patients recently admitted with acute on chronic liver failure including medical, nursing, pharmacy, dietetic and physiotherapy support
       The control arm will be standard ambulatory care following discharge including medical review and nursing coordination.
14. Key inclusion/exclusion criteria

Inclusion:

- 1. Adult patients with previous or current hepatic decompensation complicated by organ failure
  2. Diagnosis of acute-on-chronic liver failure (ACLF) using the European Foundation of the Study of Liver Failure using the Chronic Liver Failure criteria (EF-CLIF) and which includes age and white cell count. Severity is graded according to the number of organ failures (Tables 1 & 2)(17).
  3. Requiring consultation from ≥3 allied health clinicians (Table 3)

Exclusion

- 1. Not meeting EF-CLIF ACLF criteria
  2. Greater than grade 2 hepatic encephalopathy
  3. Severe chronic extrahepatic disease
  4. Human immunodeficiency virus (HIV) or ongoing immunosuppressive treatments
  5. Admission for scheduled procedure or treatment
  6. Moribund or receiving end-of-life care
  7. Active malignancy including hepatocellular carcinoma
  8. Receiving regular albumin infusions for treatment of chronic hepatorenal syndrome (excluding those for periprocedural circulatory support following large volume paracentesis)
  9. Refractory ascites managed with a intra-peritoneal catheter in-situ
  10. Inability to provide informed consent

1. Residing outside the local hospital service catchment or deemed ineligible for home visits due to staff safety or occupational hazard concerns
   1. Residing in a residential aged care facility
2. Study type
   1. Interventional
   2. Randomised controlled trial
   3. Allocation concealment mechanism and sequence generation
3. First enrolment: TBA
4. Sample size
   1. Projected: 94
   2. Current: 0
5. Recruitment status: open for recruitment
6. Primary outcome
   1. Primary composite outcome: death, waitlisting for liver transplantation or 30-day readmission from day 28 from randomisation.
7. Key Secondary Outcomes
   1. Changes in liver disease severity
   2. Quality of life
   3. Cost-effectiveness compared to standard care.
8. Ethics Review
   1. Approved
   2. Monash Health Human Research Ethics Committee
      QA/76264/MonH-2021-265874(v1)
9. Completion date
   1. Ongoing
10. Summary Results
    1. TBA
11. IPD sharing statement?
    1. Plan to share IPD: No
