# Supplementary material for: A home-based, multidisciplinary liver optimisation programme for the first 28 days after an admission for acute-on-chronic liver failure (LivR well): a study protocol for a randomised controlled trial
Source: Trials. 2022 Sep 5;23:744. doi: 10.1186/s13063-022-06679-x (PMC9444080; doi:10.1186/s13063-022-06679-x)
Supplement: Supplementary file 3 — Additional file 3. LivR Well standardised admission checklist. [file 13063_2022_6679_MOESM3_ESM.pdf]

28 Day LivR Well Program

Date____________ Time________________
Primary diagnosis: _____________________________________
Transport: □ Friend / Relative □ Taxi voucher

Day 1 of LivR Well is day after transfer to HITH – 28 days starts then
1st HITH visit is next available after transfer

**Service**	**Requirements**	**Instructions**	⎫
Nursing	Home visits *(min twice per week, up to daily if HE + requires supervised lactulose)*	Frequency =	
	FBE, UEC, LFT, Ca/Mg/Phos, INR (*Use pre filled slip)*	Frequency =<br>Day:	
	Weight measurement	Current =<br>Reportable =	
	Daily stool chart		
	Cleaning/personal care/shopping		
	Meal delivery		
	Taxi Vouchers		
Dietetics	*Indications: sarcopenia, diabetes, alcohol dependence, MUST ≥2*		
	Enteral tube feeding		
	Low salt high protein education		
	Late night snack education		
	Diabetic diet education		
	Nutritional supplements<br>*(Supply whole in first week)*	Frequency/day =<br>Flavour:	
Pharmacy	*Indications: polypharmacy (≥5 meds), diuretic/lactulose titration*		
	Medication reconciliation		
	Blister pack and local pharmacy		
	Lactulose titration/education		
Physiotherapy	*Indications: sarcopenia, fall within last 6 months, FRAT score >11*		
	Home based exercise program (standard for all LivR Well patients as described on contract)		

28 Day LivR Well Program

**Service**	**Requirements**	**Instructions**	⎫
Addiction Medicine	Consent for referral		
Neuropsychiatry	Concern from medical team regarding cognition		
Social work	*Indication: requiring long term home support services, established disability, age >65 years*		
	Initial assessment		
	Financial review		
	Council help, Aged Care Assessment, National Disability Insurance Scheme (NDIS)		
Dental	Extractions /Dentures	*Referral to be made by gastroenterology team*	
Occupational Therapy	In-clinic functional assessment		
Behaviour contract	Only if concerns regarding behaviour prior to discharge	*Completed by gastroenterology team if required*	
Medical	Weekly review by Complex Liver Care - Thursdays		

Hospital in the Home	Assessment Date	
	HITH Transfer Date (*Day 1 of 28 day LivR Well program = day after HITH transfer, first HITH visit to be **arranged as soon as possible** after transfer)*	
	Day 1 (or closest to) visit date (inc. day 1 questionnaires)	
	Week 6 (day admit, inc. bloods, questionnaires, supps if required taxi voucher if required) date	
	Week 12 (day admit, inc. bloods questionnaires, supps if required taxi voucher if required) date	

Signature _________________________ Surname ___________________________

Delegation _______________________________ Date ________________________
