# Supplementary material for: A home-based, multidisciplinary liver optimisation programme for the first 28 days after an admission for acute-on-chronic liver failure (LivR well): a study protocol for a randomised controlled trial
Source: Trials. 2022 Sep 5;23:744. doi: 10.1186/s13063-022-06679-x (PMC9444080; doi:10.1186/s13063-022-06679-x)
Supplement: Supplementary file 4 — Additional file 4. PICF. [file 13063_2022_6679_MOESM4_ESM.pdf]

Participant Information Sheet/Consent Form
Interventional Study - *Adult providing own consent*

Monash Health

**Title**	The Virtual Hospital: A digital health ecosystem supporting mobile person-centred care for chronic conditions
**Short Title**	The Virtual Hospital
**Principal Investigator**	Dr Suong Le
**Location**	Monash Health<br>Monash Medical Centre, 246 Clayton Road, Clayton VIC 3168

Part 1: What does my participation involve?

1 Introduction

You are invited to take part in a project that aims to compare a digital health intervention with current management for adult patients admitted for chronic liver disease.

This Information Sheet/Consent Form outlines the components of the study.

Please ask questions about anything that you don't understand or want to know more about. Participation is voluntary. If you don't wish to take part, you don't have to. You will receive the best possible care either way.

If you decide to take part, you (and/or your next-of-kin/guardian) will be asked to sign the consent section. By signing it you are telling us that you:

- Understand what you have read
- Agree to take part in the research project
- Agree to have the tests and follow-up described
- Agree to use of your personal and health information to assess health outcomes

You will be given a copy of this Participant Information and Consent Form to keep.

2 What is the purpose of this research?

The purpose of this study is to develop and evaluate an integrated patient management model (a communication technology platform) to improve current management for adult patients admitted for chronic liver disease.

The aims of this study are to:

- Evaluate the transition of care from inpatient to community based outpatient care
- Compare the current outpatient care with the remotely monitored outpatient model of care
- Assess and compare the quality of life of each group

- Assess and evaluate the rate of hospital admissions and healthcare resource utilisation between the groups

3 What does participation in this research involve?

This is a randomised study; you cannot choose which group you are in. If you agree to take part in this study and meet all of the requirements, you will be randomised to one of the following two management groups:

Group A: Virtual Hospital management model
Group B: Current clinical management model

Randomised means the study group you are assigned will be chosen by chance, like flipping a coin to receive one of the two management groups. You will have a 50% chance of continuing to receive current clinical management or a 50% chance of receiving management via the Virtual Hospital model.

It is desirable that your local doctor be advised of your decision to participate in this research project. If you have a local doctor, we strongly recommend that you inform them of your participation in this research project.

Item of study	Subset	Features
**Health questionnaire**	General information	• Date of birth, gender, education, job
	General Health	• History of liver conditions<br>• Previous liver treatments<br>• Risk factors for liver issues<br>• Medical conditions and medications
	Lifestyle	• Exercise<br>• Smoking<br>• Alcohol
	Health Attitudes	• Feelings about general health<br>• Attitudes and feelings about treatment<br>• Quality of life survey
**Examination**	General	• Height and weight<br>• Waist circumference<br>• Blood pressure
	Liver	• Assess for complications of liver conditions (if known/suspected)
**Blood tests** *(if not collected previously)*	Liver	• Assess for liver inflammation
	Blood viruses	• Assess for blood viruses
	Future workup	• We will store some serum (a blood component) for future studies to better understand liver health

There are no costs associated with participating in this research project, nor will you be paid.

4 What do I have to do?

Participants in this study will be cared for and managed by a multidisciplinary team of clinicians who will instruct patients to adopt lifestyle factors which have evidence of improving their chronic liver disease including: a low salt, high energy and high protein diet, reduction of alcohol and illicit drug use and medication compliance.

Participants can take their regular medication. The only medication(s) they should not take are those advised by the treating clinician as being contraindicated or likely to exacerbate their chronic liver disease.

Participants may be restricted from taking part in the study if they do not turn up for scheduled appointments or engage with the clinicians through the appropriate communication channels.

5 Do I have to take part in this research project?

You do not have to take part in this research project to receive treatment at this hospital. Other options are available; these include outpatient care through other Monash Health liver clinics or through a private Gastroenterologist. Your study doctor will discuss these options with you before you decide whether or not to take part in this research project. You can also discuss the options with your local doctor.

- Participation is voluntary. If you decide to take part, you will be given this Participant Information and Consent Form to sign and you will be given a copy to keep
- Your decision whether to take part or not will not affect your regular care, or your relationship your regular treating team

6 What are the possible benefits of taking part?

We cannot guarantee or promise that you will receive any benefits from this research, however possible benefits may include:

- Reduction in preventable hospitalisations
- Improvement in symptom control associated with chronic liver disease
- Improvement in quality of life associated with improved disease control and reduce hospitalisations

Information from this study may help inform how we can provide improved access to liver care in people admitted with chronic liver disease.

7 What are the possible risks and disadvantages of taking part?

This research does not involve any interventional treatment, procedures or any medical therapy which is not considered standard care or evidence based care for chronic liver disease.

Diagnosis of a liver condition may have insurance and potential social implications. However, the benefits of diagnosis are felt to greatly-outweigh the risks. Complications can be avoided if these conditions are treated early such as stabilisation of the liver disease, treatment of the underlying cause of the liver disease and early surveillance and monitoring for potential destabilisation or liver cancer.

8 Could this research project be stopped unexpectedly?

If treatment is commenced, there will be strong emphasis upon ensuring completion / follow-up.

9 What if I withdraw from this research project?

You can withdraw at any time (see attached form for withdrawal of participation). If you withdraw, the study team will stop collecting information. Information that has already been collected will be used for future analysis.

10 What happens when the study ends?

Depending on our conclusions from this study, the results may be published. Information will not traceable to any individuals (and will be completely de-identified). Ongoing treatment/follow-up will be provided as per standard care.

Part 2: How is the research project being conducted?

11 What will happen to information about me?

By signing the consent form you consent to the study doctor and relevant research staff collecting personal information about you for the research project. Recorded information will only be used for the purpose of this research project and will only be disclosed with your permission, except as required by law.

If you consent to involvement in this project, you will be allocated a specific code which will be connected to your Monash Health hospital identification code. Any information obtained for the purpose of this research project that can identify you, will be treated as confidential and securely stored.

Paper materials will be securely stored at Monash Health and will only be accessible by the Monash Health researchers involved in this study.

Electronic data will be entered into the secure Monash University Data Centre Restrictive Zone. The data will be entered using the specific code assigned to you and will not contain your name, address or hospital number.

The coded health information may be shared in confidence with other doctors or researchers, which may include, but is not limited to, different research institutions, other registries, hospitals, private entities, government or other health services from Australia or overseas, for research, reporting or statistical purposes, provided there is written approval by the Monash Health Human Research Ethics Committee.

The samples are referring to leftover/un-used samples of the original biospecimens. There are no plans related to residual products derived from those biospecimens. It is intended that coded data and information arising from the samples would be retained and stored securely and indefinitely in an electronic form. However, in the event the research project ceases, data and samples will be kept for a minimum of 15 years following completion of the study.

12 Access to Your Information

In accordance with relevant Australian and/or Victorian privacy and other relevant laws, you have the right to request access to your collected information. You also have the right to request that any information with which you disagree be corrected. Please contact the study team member named at the end of this document if you would like to access your information.

Your coded study information may also be used for additional unanticipated medical and/or scientific research projects in the future relating to your disease or similar diseases, but at all times in compliance with applicable law and regulation.

Any information obtained for the purpose of this research project and for the future research described in this Section that can identify you will be treated as confidential and securely stored. It will be disclosed only with your permission, or as required by law.

Stored samples from liver biopsy, blood, urine	Samples collected for any of these tests may be stored to use for additional and/ or future testing. Your stored samples will be used for research purposes only as permitted by this consent.
Future research test - OPTIONAL	If you agree, leftover blood, urine, tissue samples may be stored for future testing. If you do not agree, you can still take part in the main study. More information is below.

13 Who is organising the research?

This study will be conducted by Monash Health (Dr Suong Le) and Monash University. It is possible in the future, funding to conduct the study may be obtained from a combination of university, NHMRC and industry partners (such as commercial companies involved in production of medical equipment), and any funding obtained will be administered via Monash University.

14 Who has reviewed the research project?

All research in Australia involving humans is reviewed by an independent group of people called a Human Research Ethics Committee (HREC). The ethical aspects of this research project have been approved by the HREC of Monash Health.

This project is carried out according to the *National Statement on Ethical Conduct in Human Research (2018)*. This statement has been developed to protect the interests of people who agree to participate in human research studies.

15 Further information and who to contact.

Medical Concerns
For further information concerning this project or if you have any medical problems which may be related to your involvement in the project, please contact:

**Name**	Dr Suong Le
**Position**	Principal Investigator / Medical Consultant
**Telephone**	0413227364
**Email**	Suong.le@monashhealth.org

After Hours at Monash Medical Centre: 03 9594 6666 – Request the Gastroenterology Registrar.

Complaints contact person
If you have any complaints about any aspect of the project, the way it is being conducted or any questions about being a research participant in general, then you may contact:

**HREC Manager**	Ms Deborah Dell
**Telephone**	03 9594 4611
**Email**	deborah.dell@monashhealth.org

Reviewing HREC approving this research and HREC Executive Officer details

**Reviewing HREC name**	Monash Health Human Research Ethics Committee
**HREC Executive Officer**	Ms Deborah Dell
**Telephone**	03 9594 4611
**Email**	deborah.dell@monashhealth.org

Local Research Governance Office

**Name**	Mr Michael Kios
**Position**	Manager, Research Governance
**Telephone**	03 9594 4606
**Email**	michael.kios@monashhealth.org

Monash Health

Consent Form

SHF00057

**Title**	The Virtual Hospital: A digital health ecosystem supporting mobile person-centred care for chronic conditions
**Principal Investigator**	Dr Suong Le
**Location**	Monash Health<br>Monash Medical Centre, 246 Clayton Road, Clayton VIC 3168<br>[illegible]

Declaration by Participant / Guardian

I have read the Participant Information Sheet or someone has read it to me in a language that I understand. I understand the purposes, procedures and risks of the research described in the project.

I understand that all collected information will remain confidential. I have had an opportunity to ask questions and I am satisfied with the answers I have received.

I freely provide my consent for participation in this research project and understand that withdrawal from the follow-up is permissible at any time during the study without affecting future health care. A signed copy of this document will be provided for my/the patient's personal records.

Name of Participant (please print) ______________________________

Signature ______________________ Date ______________________

Name of Guardian (if participant unable to consent) ______________________________

Signature ______________________ Date ______________________

Declaration by Study Doctor/Senior Researcher†

I have given a verbal explanation of the research project, its procedures and risks and I believe that the participant has understood that explanation.

Name of Study Doctor/Senior Researcher† (please print) ______________________________

Signature ______________________ Date ______________________

† A senior member of the research team must provide the explanation of, and information concerning, the research.

Optional Future Research Consent

**Title**	The Virtual Hospital: A digital health ecosystem supporting mobile person-centred care for chronic conditions
**Principal Investigator**	Dr Suong Le
**Location**	Monash Health<br>Monash Medical Centre, 246 Clayton Road, Clayton VIC 3168

You are being asked to take part in future research. If you decide to not take part in this optional future research, you can still take part in the main study. This research may help scientists to better understand:

- How your disease and related diseases work
- The safety and efficacy of the Virtual Hospital versus the current standard care
- How to minimise readmission or death of patients after discharge from hospital
- Who could benefit from the new e-model of outpatient care

The results of the tests done on your blood, urine, and tissue samples (also called biologic samples) will not be given to you or your study doctor. Information from these tests may be printed in a medical journal or presented at scientific meetings. Only a summary of data from all participants will be used.

The results of this research may lead to an improved treatment, prevention or confirmation of disease. You understand and agree that by consenting to the storage and testing of your samples for possible future research, you authorise the use of your sample, the by-products of the sample, and any products developed from the sample as described by this form.

If you decide you no longer want to take part in this future testing of your biologic sample(s), your unused sample(s) will be destroyed. We may continue to use and disclose the results from samples that were tested before you withdrew your consent.

If you decide to no longer take part in the main study or are taken off the main study by your study doctor, the biologic samples you provided for future research will still be kept and may be used for future testing. If you decide you no longer want to take part in this future testing, then your unused sample(s) will be destroyed.

For this study, you are being asked to store and use the samples listed below for future testing. You may request that your stored samples be destroyed at any time by writing to the study doctor at the address listed on the first page of this form.

Carefully read the sentences below and think about your choice(s).

Check the 'Yes' or 'No' box and initial next to your choice.

Store and use your **leftover** blood, urine, and tissue samples collected during this study for future research **outside of the main study.** Your samples may be stored and used for up to 15 years after the completion of the study.

I agree to allow my leftover biologic samples to be stored after the main study testing is complete and used for future research outside of the main study.

☐ Yes _______ *(initial)* ☐ No _______ *(initial)*

Form for Withdrawal of Participation

**Title**	The Virtual Hospital: A digital health ecosystem supporting mobile person-centred care for chronic conditions
**Principal Investigator**	Dr Suong Le
**Location**	Monash Health<br>Monash Medical Centre, 246 Clayton Road, Clayton VIC 3168

Declaration by Participant

I wish to withdraw from participation in the above research project and understand that such withdrawal will not affect my routine treatment, my relationship with those treating me or my relationship with Monash Health.

Name of Participant (please print) ______________________________

Signature ______________________ Date ______________________

In the event that the participant's decision to withdraw is communicated verbally, the Study Doctor/Senior Researcher will need to provide a description of the circumstances below.

Declaration by Study Doctor/Senior Researcher†

I have given a verbal explanation of the implications of withdrawal from the research project and I believe that the participant has understood that explanation.

Name of Study Doctor/Senior Researcher† (please print) ______________________________

Signature ______________________ Date ______________________

† A senior member of the research team must provide the explanation of, and information concerning, withdrawal from the research project.

Note: All parties signing the consent section must date their own signature.
